# Predictors of stroke literacy among African Americans in the “buckle of the stroke belt”

**DOI:** 10.3389/fstro.2024.1331085

**Published:** 2024-02-27

**Authors:** N. Abimbola Sunmonu, Angela M. Malek, Carolyn Jenkins, Hyacinth I. Hyacinth

**Affiliations:** ^1^Department of Neurology, Yale University School of Medicine, New Haven, CT, United States; ^2^Department of Public Health Sciences, Medical University of South Carolina, Charleston, SC, United States; ^3^College of Nursing, Medical University of South Carolina, Charleston, SC, United States

**Keywords:** African Americans, stroke, stroke belt, health disparities, knowledge and awareness, health literacy, population health

## Abstract

**Background:**

Stroke is associated with racial disparities in morbidity and mortality and stroke outcomes. Stroke literacy is a significant predictor of on-time arrival to the emergency room for acute stroke treatment. In this study, we examined sociodemographic and socioeconomic factors that predict key aspects of stroke literacy: knowledge of stroke signs/symptoms and intent to call 911 in the event of a stroke.

**Methods:**

We analyzed archived data from a survey of African American adults over 18 years residing in the “buckle of the stroke belt.” Participants were ranked into 2 categories: low or no and moderate to adequate stroke knowledge. Then we performed univariate and multivariable analyses to determine the independent predictors of (1) knowledge of stroke signs and symptoms and (1) intent to call 911.

**Results:**

Participants aged 18–39 years (OR = 0.46, 95% CI: 0.27– 0.80) were more likely to correctly recognize stroke signs and symptoms compared to those who are 65 years and above. Those age 40–64 years were also more likely to recognize stroke signs and symptoms compared to those who are 65 years and above. On the other hand, those with less than high school (OR = 2.83, 95% CI: 2.03–3.96) or complete high school education (OR = 1.95, 95% CI: 1.28–2.96) were less likely to recognize stroke signs and symptoms. Males were less likely (OR = 0.65, 95% CI: 0.64–0.66) to report that they would call 911 in the event of a stroke. While respondents aged 40–64 years (OR = 1.87, 95% CI: 1.14–3.09) and those with moderate to adequate knowledge of stroke (OR = 1.39, 95% CI: 1.18–1.65) were more likely to call 911 in the event of a stroke. Socioeconomic status was generally associated with stroke literacy.

**Conclusion:**

Among resident of the “buckle of the stroke belt,” we observed that age, sex, and educational level are among the key predictors of knowledge of stroke signs and symptoms and intent to call 911 in the event of a stroke. Stroke literacy and educational programs needs to incorporate these key sociodemographic aspects as a strategy for improving literacy and reduce stroke-related disability and health disparities.

## Introduction

Stroke is the 5th leading cause of death in the US and a significant cause of health disparities, disability, lost productivity, and healthcare costs (Go et al., 2013; Ovbiagele et al., 2013). About 795,000 strokes occur annually in the US; two-thirds are recurrent (Ovbiagele et al., 2013). By 2030, a projected 4 million more Americans will have had a stroke — a 21.9% increase over current prevalence (Heidenreich et al., 2011). African Americans and Hispanics are disproportionately affected compared with White Americans (Go et al., 2013). Prevalence of childhood and adolescence strokes remains low (Fullerton et al., 2004) but is expected to rise due to increasing obesity (Singh et al., 2008; Stamatakis et al., 2010; Rendall et al., 2012) and hypertension (Liker et al., 1988) in this demography. One estimate projects that by 2030, stroke will cost the American economy over $184 billion in direct costs and $56 billion in indirect costs (primarily lost productivity) annually (Ovbiagele et al., 2013). African Americans and Hispanics have the highest burden of stroke but are also the most likely to be socioeconomically disadvantaged (Go et al., 2013). Compared with White people, African Americans are less likely to return to work even 1 year after a stroke (Busch et al., 2009). African Americans also have a disproportionately high prevalence of stroke risk factors and low stroke literacy. There is thus an imperative to identify effective health education and preventive strategies targeted to this high-risk population.

Preventing stroke and associated disability is the focus of the American Heart Association's 2020 vision for health and cardiovascular disease (CVD) (Go et al., 2013). Intravenous thrombolysis and endovascular thrombectomy shortly after symptom onset reduce acute ischemic stroke disability. Conversely, stroke prevention is highly reliant on risk factor modification by keeping individuals at a lower risk state or moving them from a higher to a lower risk state (Ruland et al., 2003; Sallar et al., 2010). Seeking urgent care for stroke symptoms and stroke risk factor mitigation are associated with health literacy. Two cornerstones of health literacy relevant to stroke are the degree of knowledge of stroke signs and symptoms among the target population (Williams and Noble, 2008; Sallar et al., 2010) and intent to call emergency services (i.e., 911) in the event of a stroke. Such stroke literacy varies by race/ethnicity, formal education, and income (Malek et al., 2014) and is independently related to risk factor modification and post-stroke outcomes. Stroke survivors may have different stroke literacy because stroke education is provided as part of their routine clinical care. Therefore, a primarily stroke-free population may better represent the average stroke literacy in the broader population. Previous studies of stroke survivors have examined predictors of stroke literacy in South Carolina (Malek et al., 2014) and other parts of the US (Stroebele et al., 2011). None has examined predictors of stroke literacy in a general, primarily stroke-free population of African American adults in South Carolina, the “buckle of the stroke-belt,” where stroke mortality is highest.

There is a disproportionally high stroke incidence, prevalence, severity and mortality among nine states in the southeastern US collectively called the “stroke belt.” South Carolina has significantly higher stroke incidence and mortality than the national average or any other state in the stroke belt. As such, it is referred to as the “buckle of the stroke belt” [Burden of Heart Disease and Stroke in South Carolina, 2006; Centers for Disease Control and Prevention (CDC), 2012]. Stroke incidence and mortality in South Carolina are significantly higher than other parts of the US (Burden of Heart Disease and Stroke in South Carolina, 2006), but stroke knowledge is among the lowest nationwide [Centers for Disease Control and Prevention (CDC), 2004; Ellis and Egede, 2008a,b; Ellis et al., 2009]. South Carolina also has a higher proportion of African Americans (28%) compared with the rest of the country (United States Census Bureau, 2012). At the time of our study, knowledge of stroke signs and symptoms in South Carolina was about 15.8% in the general population but only 9.3% in African Americans [Centers for Disease Control and Prevention (CDC), 2012]. Among other ethnic minorities, veterans, stroke survivors and other at-risk populations highly impacted by stroke in South Carolina, stroke literacy is generally suboptimal but often still higher than African Americans in particular. For example, only about 17% of Hispanics in one study recognized four primary stroke symptoms (Ellis et al., 2009; Sallar et al., 2010). Most participants confirmed they would call 911 for a stroke, but only 23% who knew four specified stroke signs would call 911 for a stroke (Ellis et al., 2009; Sallar et al., 2010). Among veterans and stroke survivors, age, race, and educational level are independent determinants of these aspects of stroke literacy (Perry and Roccella, 1998; Ellis and Egede, 2008a). Despite some demographic similarities between our study population and these other at-risk groups, it was unclear whether stroke literacy and its driving factors are also similar.

In this study, we define “stroke literacy” as knowledge of stroke symptoms and intent to call emergency services for those symptoms. We sought to answer the overarching question *What are the independent predictors of stroke literacy?* Our primary hypothesis was that respondents with more favorable demographic and economic profiles would have greater stroke literacy in the studied domains. To our knowledge, this is the first study among South Carolinians with the overall goal of identifying independent predictors of stroke literacy in a general, primarily stroke-free population of African Americans. We also evaluated the 10-year trend in stroke literacy. This study is important because stroke literacy is crucial in early recognition of stroke, which is itself critical for early arrival to the emergency room and access to life saving treatment (Bohannon et al., 2003). Our findings further illuminate important considerations in the development of targeted educational campaigns in South Carolina and the stroke belt.

## Materials and methods

### Study design and data collection

This is a quantitative longitudinal cross-sectional study design utilizing secondary data analysis. Data sources are REACH 2010 and REACH US (Racial and Ethnic Approaches to Community Health, later renamed Racial and Ethnic Approaches to Community Health Across the United States; collectively referred to as REACH in this paper) surveys of the Centers for Disease Control and Prevention conducted at the Medical University of South Carolina (MUSC). REACH aims to reduce racial and ethnic health disparities across a wide range of health issues. REACH focused on Charleston and Georgetown counties in South Carolina, and was carried out via English-language mailed questionnaires and telephone interviews (Bruce et al., 2008). This study reports aggregate anonymized data from 2003 to 2012.

The five stroke symptoms presented to participants are (1) sudden confusion or difficulty speaking; (2) sudden numbness or weakness of the face, arm, or leg, especially on one side; (3) sudden difficulty seeing in one or both eyes; (4) sudden difficulty walking, dizziness, or loss of balance; and 5) severe headache without known cause. Correctly identifying 1 or no stroke symptom was classified as low or no knowledge, while correctly identifying 2 or more symptoms was classified as moderate or adequate knowledge (Supplementary Table 3). We examined the yearly proportion of individuals with adequate and low health literacy. We also determined the year-to-year proportion of individuals who said they would call 911 for a stroke.

### Study population

The population was entirely composed of respondents to the REACH survey conducted in South Carolina. They were English-speaking African American adults living in Charleston and Georgetown counties.

### Study variables

Two dimensions of stroke literacy were chosen as the main dependent variables: knowledge of stroke signs and symptoms, and intent to call 911. Previous studies of stroke literacy have also focused on one or more of these, therefore facilitating comparisons and contextualization of the present study. Major independent variables are socio-demographic factors such as age, race and sex; economic factors such as education and income; and knowledge of signs and symptoms. Relevant covariates analyzed include history of stroke, past or current history of a medical condition predisposing to stroke, and history of cigarette smoking. Level of knowledge of stroke signs and symptoms was derived from adding correct recognition of any of 5 stroke symptoms (correct response = 1, incorrect response = 0).

### Statistical analyses

The sampling strategy was based on a random stratified cluster sampling approach. Participants were weighted to ensure that the analytical results were representative of the entire state of South Carolina (Frankfort-Nachmias and Nachmias, 2008a). The sampling strategy was previously detailed (Hyacinth, 2015). We reviewed raw data to ensure appropriate coding of variables. Then we conducted a descriptive analysis to characterize respondents. Next, we performed univariate, unadjusted and subsequent multivariable (logistic regression) analyses to identify independent predictors of our chosen metrics of health literacy. The cross-sectional survey design provides a snapshot of potential determinants of knowledge and appropriate action in the event of a stroke (Frankfort-Nachmias and Nachmias, 2008b,c).

Because this was a cross-sectional study conducted annually and not a longitudinal study over multiple years, missingness was not problematic. We assumed missingness due to non-return of the questionnaire occurred at random. Returned surveys with missing data were addressed with follow-up phone calls to complete the responses. Missingness also arose if only specific participants were required to respond, e.g., questions pertaining only to people with diabetes. Accounting for these scenarios, the remaining missingness was minimal and assumed to be at random, and the analyses were completed without additional modifications.

### Definitions

We measured the level of *stroke knowledge* by the number of stroke symptoms named by the respondent. *Intent to call 911* was derived from a categorical response to the question of what the respondent would do if they were having a stroke or witnessed someone having a stroke. *Educational level* was used as a surrogate for academic literacy and defined by completion of a specific level of formal education. *Income* was based on US Census definitions. Since cardiovascular disease is strongly related to stroke, we also compared knowledge of cardiovascular disease to stroke knowledge to determine if there is any crossover relationship between health literacy in both cases.

## Results

A total of 11,537 unweighted respondents were included in the analyses. They comprise nine survey years averaging at least 800 respondents per year (exact number of years differed by variable).

### Participant characteristics

Baseline sociodemographic health behavior and other participant characteristics are summarized in Table 1 and Supplementary Tables 1, 2. Most respondents were female, aged 40–64 years and responded by telephone.

**Table 1:** Socio-demographic variables and survey characteristics.

**Variable**	**Weighted count**	**Un-weighted count**	**Percent (%)**	**95% CI of percent (%)**
**Mode of questionnaire administration**				
In person	49.3	40	0.5	0.1–4.8
By telephone	7564.2	7,568	77.9	63.9–87.5
By mail	2100.4	2,106	21.6	11.4–37.1
**Age category (years)**				
18–39	1703.6	838	40.5	40.0–41.0
40–64	1869.8	2,249	44.4	44.3–44.6
65 and older	634.9	1,121	15.1	14.8–15.4
**Sex**				
Male	4051.4	2,973	41.7	38.8–44.6
Female	5662.3	6,740	58.3	55.4–61.2
**Born in the United States**				
Yes	4110.5	4,130	97.9	97.2–98.4
No	87.8	65	2.1	1.6–2.8
**Educational level**				
Never attended school or only kindergarten	15.5	14	0.2	0.1- 0.3
Did not complete high school (grades 1-11)	1752.6	1,814	18.1	16.9–19.4
Completed high school (12th grade or GED)	3661.2	3,496	37.9	36.6–39.1
Some college/College graduate/Graduate school	424.7	4,337	43.9	42.2–45.6
**Occupational status**				
Employed	5012	4,317	52.3	50.5–53.6
Unemployed	1355.7	1,241	14.1	13.1–15.1
Student	513.5	329	5.3	4.5–6.3
Retired/Unable to work	2720.9	3,306	28.3	26.5–30.0
**Household income**				
$25,000	4828.8	4,850	54	52.4–55.6
$25,000 to < $50,000	2733.6	2,658	30.6	29.4–31.8
$50,000 to < $75,000	773.8	832	8.7	7.9–9.5
$75,000 or more	600.5	604	6.7	5.8–7.7
**Has health insurance coverage**				
Yes	2910.7	3,190	70.1	68.2 - 72.0
No	1239.6	967	29.9	28.0–31.8
**Need but can't afford a doctor?**				
Yes	2246.9	2,134	23.3	21.2–25.5
No	7410	7,512	76.7	74.5–78.8
**Worried about funds to pay for healthy foods**				
Always/Usually/Sometimes	4101.5	4,109	98.3	97.2–99.0
Rarely/Never/Not Applicable	69.4	50	1.7	1.0–2.8
**Body Mass Index Category (kg/m** ^2^ **)**				
Normal weight	2606.7	2,378	27.7	26.9–28.6
Overweight	3126.8	3,109	33.3	32.2–34.4
Obese	3661.1	3,853	39	37.9–40.0
**Cigarette smoking**				
Current smoker (everyday)	1298.6	1,139	13.5	12.6–14.5
Current smoker (some days)	688.4	586	7.2	6.5–7.9
Former smoker	1601.3	1,789	16.6	15.7–17.7
Never smoked	6039.4	6,098	62.7	61.9–63.5

Two-thirds of participants were overweight or obese. Among respondents with diabetes, only 77% checked their blood glucose regularly within the past year. Hypertension prevalence was 44.4%, and 82.6% of affected respondents were aware that regularly taking anti-hypertensives can help control hypertension. About 37% had high cholesterol, 82.4% of whom received prescription cholesterol lowering medications. Over 70% of respondents worried about affording healthy foods. The study population was primarily stroke-free. Their stroke prevalence of 3.8% (Supplementary Table 3) was higher than a 5-year (2006–2010) average prevalence of 3.0% in South Carolina and 2.6% nationally (Burden of Heart Disease and Stroke in South Carolina, 2006). Compared with the general population, the proportions of smokers and obese or overweight individuals were similar but slightly lower at 20.7% (Jacobs et al., 2015) and 70% (Ogden et al., 2014), respectively.

Notably, 54.0% had household incomes below $25,000. About half of those who reported their household income level lived below the poverty level of < $25,000 annual household income for a family of 4. Despite 70% of respondents being insured, 23% said they could not afford a doctor even when they needed one. Weighted and un-weighted data were overall quite similar but more different for sex and least for income. Employment level was significantly below national average.

### Stroke literacy

Regarding stroke symptoms, 90% of respondents correctly identified numbness or weakness of the face, arm and leg, and 82.2% correctly identified sudden confusion or trouble speaking (Supplementary Table 3). Only 53.5% correctly recognized sudden trouble seeing in one eye as a stroke symptom. Surprisingly, 42.5% of respondents incorrectly identified chest pain and discomfort as a stroke symptom. Trouble walking, dizziness or loss of balance were correctly identified by 77.6%, and severe headache by 54.6%. Overall, 86.1% of respondents had moderate to adequate knowledge of stroke signs. Furthermore, 88.6% said they would dial 911 were they to witness someone having a stroke or heart attack. Only 20.9% had heard of a cardiovascular or cerebrovascular disease prevention program being conducted or available in their area. Detailed results are presented in Supplementary Table 3.

Overall, knowledge of stroke symptoms was associated with education, household income level, age of respondents, employment status, obesity risk factors, respondent's management of diabetes, hypertension status, dietary habits, and even recognition of symptoms of other cardiovascular disease. In turn, knowledge of symptoms and signs of both cardiovascular and cerebrovascular disease was a significant independent predictor of intent to call 911 in the event of either.

### Univariate analyses of stroke literacy

*Socio-demographic characteristics:* Younger respondents aged 18–39 and 40–64 years old were more likely to have moderate to adequate stroke knowledge compared with those 65 years or older (Table 2). Compared to respondents with college or higher education, those who never attended school, had between 1st and 11th grade education, or completed high school were significantly more likely to have low to no knowledge of stroke symptoms. Employed, unemployed and student respondents were more likely to have moderate to adequate knowledge of stroke symptoms compared with respondents who were retired or unable to work. An annual household income of <$25,000 and $25,000 to <$50,000 was associated with low to no knowledge of stroke symptoms compared with respondents reporting an annual household income of $75,000 or greater. Finally, respondents whose self-reported height and weight put them in an obese category were 21% more likely to have low to no knowledge of stroke signs (Table 2).

**Table 2:** Univariate relationship between level of knowledge of stroke signs and symptoms and basic demographic characteristics of respondents.

**Variable**	**Odds ratio**	**95% confidence interval**
**Mode by which questionnaire was administered**		
In person	0.41	0.37–0.48
By telephone	1.1	0.86–1.39
**Age category (years)**		
18–39	2.57	2.17–3.06
40–64	2.31	1.44–3.71
Sex	1.15	0.89–1.49
Born in the United States	0.9	0.32–2.55
**Educational level**		
Never attended school or only kindergarten	17.12	3.12–93.97
Did not complete high school (grades 1-11)	4.87	4.08–5.80
Completed high school (12th grade or GED)	2.56	2.16–3.09
**Occupational status**		
Employed	2.56	2.29–2.86
Unemployed	1.7	1.31–2.20
Student	2.27	1.38–3.75
**Household income**		
$25,000	3.46	2.18–5.76
$25,000 to < $50,000	2.28	1.32–3.94
$50,000 to < $75,000	1.02	0.44–2.38
Has health insurance coverage	0.82	0.59–1.14
Need but can't afford a doctor?	1.17	0.87–1.57
Worried about funds to pay for healthy foods	0.67	0.13–3.53
**Body Mass Index Category (kg/m** ^2^ **)**		
Normal weight	0.81	0.67–0.99
Overweight	0.93	0.69–1.25
**Cigarette smoking**		
Current smoker (everyday)	0.9	0.76–1.08
Current smoker (some days)	0.69	0.47–1.01
Former smoker	1.04	0.84–1.29

*Health behavior and health characteristics:* Participants were significantly more likely to have moderate to adequate knowledge of stroke symptoms if they reported their health status as excellent, very good, good or fair; reported zero or ≤7 days of poor physical health in the preceding month; or had a foot or eye exam in the preceding year (Supplementary Table 4). Similarly, respondents were less likely to have low stroke literacy if they had hemoglobin HbA1c testing in the prior year, were physically active outside of work, had their cholesterol checked or avoided high fat or high cholesterol diets.

*Medical history, risk factor profile and management:* Low to no knowledge of stroke symptoms was more likely among participants previously diagnosed with diabetes mellitus or hypertension and less likely among those who reported having taken a course on diabetes self-management (Supplementary Table 5). Low to no stroke literacy was less likely among respondents who reported being advised to eat less fatty or high cholesterol foods.

*Cardiovascular literacy and relationship to stroke literacy:* Individuals with a history of myocardial infarction or who correctly identified its symptoms in general, and especially arm or shoulder discomfort and shortness of breath, were more likely to have moderate to adequate stroke literacy (Supplementary Table 6). Those taking aspirin to lower heart attack or stroke risk were less likely to have low to no stroke literacy. Respondents who incorrectly identified trouble seeing in one or both eyes as a symptom of heart attack were significantly more likely to have low to no health literacy. Overall, moderate to adequate knowledge of symptoms of heart attack was associated with a higher likelihood of being knowledgeable about stroke symptoms. Finally, moderate to adequate stroke literacy was associated with a higher likelihood of calling 911 for stroke or heart attack symptoms.

### Multivariate analyses to identify determinants of stroke literacy

Educational level was a significant independent predictor of stroke knowledge (Table 3). Compared to those with college level or higher education, respondents with a high school education or lower were more likely to have low to no knowledge of stroke symptoms. However, educational level was not a significant predictor of intent to call 911 (Table 4).

**Table 3:** Multivariate analyses to identify independent predictors of level of knowledge of stroke signs and symptoms.

**Variable**	**Odds ratio**	**95% confidence interval**
**Educational level**		
Some college/College graduate/Graduate school	1.00 (ref)	
High school graduate (12th grade or GED)	1.95	1.28–2.96
Did not complete high school	2.83	2.03–3.96
Never attended school or only attended kindergarten	—	—
**Annual household income**		
$75, 000 or more	1.00 (ref)	
$50, 000 to < $75, 000	1.18	0.12–11.65
$25, 000 to < $50, 000	2.14	0.34–13.29
$25, 000	3.76	0.64–22.05
**Age category**		
65 years and older	1.00 (ref)	
40–64 years	0.53	0.27–1.02
18–39 years	0.46	0.27–0.80
**Sex**		
Female	1.00 (ref)	
Male	1.08	0.68–1.72

**Table 4:** Multivariate regression analysis to determine the independent predictors of intent to call 911 in the event of a stroke.

**Variable**	**Odds ratio**	**Confidence interval**
**Educational level**		
Some college/College graduate/Graduate school	1.00 (ref)	
High school graduate (12th grade or GED)	1.16	0.69–1.98
Did not complete high school	1.03	0.80–1.33
Never attended school or only attended kindergarten	—	—
**Annual household income**		
$75, 000 or more	1.00 (ref)	
$50, 000 to < $75, 000	0.98	0.63–1.54
$25, 000 to < $50, 000	0.59	0.23–1.51
$25, 000	0.63	0.26–1.49
**Age category**		
65 years and older	1.00 (ref)	
40–64 years	1.87	1.14–3.09
18–39 years	1.41	0.69–2.90
**Sex**		
Female	1.00 (ref)	
Male	0.65	0.64–0.66
**Categorical level of knowledge of stroke**		
Low or no knowledge	1.00 (ref)	
Moderate to adequate knowledge	1.39	1.18–1.65

Compared to a reported annual household income of >$75,000, annual household incomes of <$25,000, $25,000 to <$50,000 or $50,000 to <$75,000 correlated with low to no knowledge of stroke signs (Table 3). Although the level of knowledge worsened with lower annual household incomes, this difference was not statistically significant. Similarly, household income was not a statistically significant predictor of intent to call 911 for a stroke or heart attack.

Participants aged 18–39 years were more likely to correctly recognize stroke signs and symptoms (OR = 0.46, 95% CI: 0.27–0.80). However, age 40–64 years was more predictive of stroke literacy (OR = 0.53, 95% CI = 0.27–1.02) when compared with age 65 and above. Intent to call 911 for a stroke or heart attack was greater in those aged 40–64 years. Males were more likely to have low to no knowledge of stroke signs, but this difference was not statistically significant. Males were also less likely (0.65, CI = 0.64–0.66) to call 911 in the event of a stroke or heart attack. Level of knowledge of stroke symptoms significantly predicted intent to call 911. Moderate to adequate knowledge was associated with a higher likelihood of intent to call 911 for a stroke. There was a significant upward yearly trend in the level of knowledge and intent to call 911 (*p* < 0.001) (Table 5). Except for 2005, there was also a steady year-to-year (2001–2012) increase in the proportion of participants with moderate to adequate stroke literacy.

**Table 5:** Proportion of respondents by year with moderate to adequate knowledge of stroke and/or intent to call 911 in the event of a stroke.

**Year (*n*)**	**Knowledge of signs and symptoms (%)**		**Action in the event of a stroke (%)**	
	**Moderate to adequate**	**Low to none**	**Call 911**	**Do something else**
2001 (921)	82.2	17.8	84.9	15.1
2003 (901)	82.3	17.7	85.8	14.2
2004 (1850)	84.8	15.2	87.4	12.6
2005 (927)	81	19	86	14
2006 (903)	88	12	88	12
2009 (908)	90.3	9.7	89.6	10.4
2009-2010 (1268)	88.7	11.3	91.5	8.5
2010-2011 (1031)	88.8	11.2	92.3	7.7
2012 (1001)	88.5	11.5	91.6	8.4

## Discussion

### Key findings

In this study, we examined the sociodemographic factors that are associated with stroke knowledge and intent to call 911. It is worth nothing that is one of the largest studies of its kind among individuals residing in the “buckle of the stroke belt.” We noted that several socio-demographic, economic and lifestyle factors such as, knowledge of stroke symptoms was associated with education, household income level, age of respondents, employment status, obesity risk factors, respondent's management of diabetes, hypertension status and dietary habits, were associated with stroke literacy. In turn, knowledge of symptoms and signs of both cardiovascular and cerebrovascular disease was a significant independent predictor of intent to call 911 in the event of either. It was worrisome that several respondents confused the signs and symptoms of stroke and heart attack. For instance, 31% thought trouble seeing in one or both eyes symptomized heart attack while 42.5% thought chest pain or discomfort was a symptom of stroke. While the response in both cases may be to call 911, it is possible that inappropriately ascribing symptoms of a stroke to another medical problem may diminish the urgency with which emergency help is sought. Lifestyle and cardiovascular disease history also significantly predicted their stroke literacy. In multivariate analyses, age and educational level were the most significant independent predictors of stroke literacy. Age, sex and knowledge of stroke signs and symptoms were significant independent predictors of intent to call 911 for a heart attack or stroke. There was a significant upward trend in both measures of stroke-related health literacy over time.

### Interpretation of findings

Prevention of stroke, stroke-associated disability and mortality is reliant on risk factor modification and acute interventions. Any successful educational campaign must not only improve the knowledge of stroke symptoms but also increase the number of patients potentially eligible for acute interventions. Here, we investigated the factors that predict key components of stroke literacy—knowledge of stroke symptoms and intent to call 911—in South Carolina, focusing on African Americans. We also determined the trend in health literacy. These insights can provide actionable information for designing targeted stroke education campaigns and evaluating the impact of existing programs. Assertions by some previous studies that level of stroke knowledge does not improve intent to call 911 (Mikulík et al., 2011; Skolarus et al., 2013) also necessitated investigation. Ascertaining whether aspects of cardiovascular health literacy interact with stroke literacy provided further insights into the latter.

This study is the largest in scale among comparable studies and the first of its kind in the non-veteran African American population in South Carolina. Overall, our findings agree with several similar studies, while they stand in contrast to others that have examined the same or similar questions in a different population and geographical region of the Unites States. Between 2001 and 2005, the proportion of participants with moderate to adequate knowledge of stroke in our study ranged from 81.0% to 84.8%. Except for 2005 (when it fell from 84% to 81%), there was a steady yearly increase in this dimension of stroke literacy. Both findings contrast sharply with another study performed elsewhere in the US, in which knowledge of stroke symptoms over several years was as low as 48% and then plateaued at 68% from 2000 to 2005 (Kleindorfer et al., 2009). A previous REACH study in South Carolina showing only 15.8% stroke literacy in general and 9.3% in African Americans had more stringent criteria for stroke literacy than our study that could account for the apparent discrepancy [Centers for Disease Control and Prevention (CDC), 2012]. Notably, participants were deemed stroke literate only if they correctly identified 5 signs of stroke, excluded chest pain or discomfort as stroke symptoms, and indicated intent to call 911 for any stroke symptoms [Centers for Disease Control and Prevention (CDC), 2012].

Education was associated with level of knowledge of stroke signs and symptoms. Compared with respondents with college level or higher education, those who attained lower education levels were more likely to have low or no knowledge of stroke symptoms. This mirrors previous studies among two other stroke-vulnerable groups in South Carolina: Hispanics and military veterans. In those studies, Hispanic respondents with high English literacy skills and veterans with high school or higher level of education were significantly more likely to accurately recognize two or more signs of stroke (Ellis and Egede, 2008a,b; Ellis et al., 2009).

About 88% of respondents in this study would call 911 for a stroke or heart attack. This ranged from a low of ~85% in 2001 to a high of 92% in the 2010–2011 survey. It was associated with a steady rise in knowledge of stroke signs and symptoms. In a similar study among patients with a previous stroke, the proportion of African American respondents who would call 911 was 81.35% (Ellis and Egede, 2008b). One study showed no improvement in the intent to call 911 after a 4 year education campaign, based on a stroke action test score (STAT) that was 18% at baseline and 19% after the intervention (Mikulík et al., 2011). In a study of 108 people hospitalized with acute ischemic stroke in South Carolina, the likelihood that they called 911 for their stroke was highest if they recognized their symptoms as stroke symptoms, had prior experience with stroke (self or a loved one), or prior experience calling emergency medical services (Xirasagar et al., 2019). Conversely, they were least likely to call 911 if they were discouragement by bystanders or called their physician's office at symptoms onset and were advised to do anything else (Xirasagar et al., 2019).

Socioeconomic status is determined in large part by education and household income. In this study, household income did not predict stroke literacy, although education level did. Since education is also a partial determinant of income, i.e., higher educational level correlates with higher income and therefore socioeconomic status, the relationship between socioeconomic status as a whole and stroke literacy may reflect the strength of association of its individual components in complex ways that we cannot determine from the data available in this study.

The present study demonstrates better stroke literacy in people who previously had a stroke, but a small study of stroke literacy in 134 male stroke survivors in South Carolina using the Stroke Knowledge Test showed significant knowledge gaps in this population (United States Census Bureau, 2012). There was no difference across race when adjusted for age, education, and marital status (United States Census Bureau, 2012). Nationally, stroke literacy in stroke survivors is also low, especially in African Americans. In a group of 2,970 adults nationwide with previous stroke, fewer than 35% could distinguish all 5 signs of a stroke provided and would call 911; this dropped to 22.3% among non-Hispanic African Americans (Ellis and Egede, 2008b).

In our study, the independent determinants of moderate to adequate knowledge of stroke signs were education and younger age. Those of intent to call 911 were age, sex and knowledge of stroke symptoms. Our study shows that 88% of people would call 911 for a stroke, consistent with the 90% previously reported by another group (Alkadry et al., 2005). Intent to call 911 was highest among younger respondents and lowest among older respondents, consistent with a previous study from Spain (Lundelin et al., 2012). Our findings also support multiple studies demonstrating that awareness of stroke symptoms increases the intent to call 911 for stroke (Miller et al., 2007; Williams and Noble, 2008; Mullen Conley et al., 2010; Williams et al., 2012). Nevertheless, one report found that increasing knowledge of stroke symptoms through stroke education resulted in no appreciable improvement in the intent to call 911 (Mikulík et al., 2011). Apparently contradictory reports of the relationship between stroke literacy and intent to call 911 may be due to variability in defining stroke knowledge in a way that precludes direct comparison.

### Implications

This study has important findings for public health, health policy, health economics, healthcare equity and access, and future research. The health and economic impact of stroke and the huge impact of behavior modification in prevention inform efforts to design strategies for stroke prevention (Ovbiagele et al., 2013). Culturally appropriate and relevant stroke education are likely to be more successful in improving stroke literacy (Williams and Noble, 2008; Mullen Conley et al., 2010). The National Institute for Neurological Disorders and Stroke also recommends that stroke education programs start with a community needs assessment to increase the chance of success (Daley et al., 1997). In this study, participants actively involved in managing their health were more knowledgeable of stroke signs and symptoms, suggesting that engaging people in their own healthcare can improve knowledge of stroke literacy. Individuals with a prior heart attack, angina, and other coronary heart disease symptoms more accurately recognized the symptoms of stroke. Thus, comprehensive stroke education at a hospital visit for cardiovascular disease may be impactful. Only about 21% of respondents were aware of a local health education program, suggesting the need to improve publicity for health education programs. We are concerned that lower education and older age were associated with lower stroke literacy. People with low education are less likely to have health insurance, more likely to be of low socioeconomic status and more likely to suffer a stroke. As such, we recommend that stroke education be geared toward this population.

Our findings also demonstrate the marked knowledge gap among older adults, who are already more vulnerable to suffering a stroke. Given the changing demography of the US and more children being raised by grandparents (Hayslip and Kaminski, 2005; Hank and Buber, 2009), improving stroke literacy in children and younger adults may counter low stroke literacy in older adults. Indeed, stroke education in formal settings like schools encourages adolescents and young adults to recognize stroke symptoms and be more likely to call 911 in the event of a stroke in the elderly (Morgenstern et al., 2007; Williams and Noble, 2008; Mullen Conley et al., 2010; Williams et al., 2012).

Since most respondents were worried about affording healthy foods, policies that help eliminate food deserts may also improve cardiovascular and specifically stroke risk management. Since stroke literacy was highest among individuals who were overweight or obese and thus at high stroke risk, social engineering to increase sidewalks, public workout areas (e.g., in local parks) and low-cost gym membership are also important avenues to reduce stroke risk.

Improved awareness of stroke signs and symptoms engenders increased intent to call 911. This is particularly important because it demonstrates that certain aspects of stroke literacy can reinforce others in feedback. At the individual level, this knowledge may inform optimized patient counseling in the limited time they have with providers at their clinic visits. At the population level, it can guide judicious spending of scarce resources for maximum impact on public health education. Even health policy may be guided by a richer understanding of the interplay between different facets of health literacy.

Two of our findings may be surprising to some. First, variables that accurately predict one dimension of stroke literacy may not predict another, even when both dimensions are strongly correlated. For instance, education level correlates with stroke knowledge but not intent to call 911, although stroke knowledge itself accurately predicts intent to call 911. This underscores the likelihood that each component within the composite metric of stroke literacy is uniquely influenced by individual patient factors. More research is needed to understand the interplay between each variable within the network of stroke literacy. Second, increasing household income is not associated with increased intent to call 911. This and other studies previously referenced demonstrate that the primary driver of intent to call emergency medical services is really awareness of stroke symptoms. Our study also demonstrates that household income is not significantly associated with stroke literacy. Therefore, household income is not a surrogate for health literacy or specific disease awareness. It would be erroneous to assume that wealthier households are more likely to call 911 for stroke symptoms because the primary driver for such intended action is actually stroke literacy, which does not correlate with household income. As such, there is an important need for stroke literacy efforts not just in poor communities but also within the general populace.

### Assumptions and potential limitations

In surveys involving phone and mailed questionnaires such as this, it is impossible to confirm the original responses, so it is assumed that the response is from the intended respondent or target population (Frankfort-Nachmias and Nachmias, 2008b). The use of a stratified random cluster sampling technique with weighting assumes that the sample will be representative of the state of South Carolina. Thus, this study presumes that responses are representative of the African American population of South Carolina. But research participation is voluntary, individuals of lower socioeconomic, and educational status are often less likely to participate in research (Schneider, 2009). We also propositionally hypothesized that education, sociodemographic factors, and income are related to and therefore predict the direction and magnitude of the independent variables, but this may not be true. It is assumed that data generated will satisfy the basic requirements for the use of a statistical test, such as a normal age distribution for a *t*-test comparing mean ages (Field, 2009). Since we compared changes in the trends of the dependent variables, we assumed that the population structure is similar from year-to-year. While this assumption might be valid for the purpose of this study, it is rarely the case in real life because of migration and other effects on population structure.

Our large sample size minimizes errors and biases, but some important limitations remain. First, there are slight differences in the structure and content of the full questionnaire and questions. However, this did not apply to the questions used in this study. Second, the suggested options for responses like “yes” or “no” were alternated from question to question. This was done to ensure that respondents were thoughtful in their responses, but it is unclear how this might have affected the responses.

All the data were previously collected, making confirmation of responses from each respondent impossible. However, the robust nature of the sampling minimizes the potential for a few incorrectly recorded responses to overly sway the underlying findings and to do so consistently from year-to-year. Our exclusive focus on African Americans may limit generalization to other racial and ethnic groups. The majority of our population had a routine health visit in the year preceding the study, so these data may not be generalizable to those with infrequent or no contact with formal healthcare structures. Most participants were under 65 years, but it is unclear if and how age impacts our findings. Certain granular details were limited with this data set. Notably, we could not ascertain whether patients with a history of stroke in themselves or close loved ones were more likely to have better stroke literacy. While the data were treated longitudinally, this study was a quasi-cross-sectional longitudinal study, so some respondents may have been surveyed multiple times over years. This could have impacted their responses. Nevertheless, this likely has minimal effect on the validity of our studies because of the survey design and prior validations of similar quasi-cross-sectional studies in the Behavior Risk Factor Surveillance System (BRFSS) national surveys.

The covariates analyzed, such as history of stroke or related medical condition, could increase chances of an individual's prior exposure to stroke education. Since African Americans are disproportionately impacted by stroke and especially in the “stroke belt,” such previous exposure to stroke education might skew the data in a broader, general population if previous stroke education is over-represented in African Americans vs. other racial and ethnic groups.

Finally, these data may not reflect recent changes in stroke literacy in this population. COVID-19 has had a remarkable effect on health education curricula, spending, and implementation. The early days of the pandemic also changed behavior around stroke, such as decreasing pursuit of urgent medical attention for stroke symptoms to minimize exposure to COVID-19. It would be interesting to see if and how stroke literacy has changed in the intervening years since the data on which the current study is based. Unfortunately, there is no currently available literature with updated comparable information.

In conclusion, this, and other studies (Morgenstern et al., 2007; Mullen Conley et al., 2010; Williams et al., 2012) show that several sociodemographic factors influence knowledge of stroke signs and symptoms which can in turn influence the intent to call 911 in the event of a stroke. Therefore, understanding the determinants of these facets of stroke literacy can strengthen them with targeted education campaigns. To our knowledge, ours is the first study of its kind in South Carolina. With population level data in a large African American community, our study identified several factors that predict individual level stroke literacy. These findings are important for designing targeted health education and better focusing, of scarce health education resources. In this study, educational level and age were the independent predictors of stroke knowledge. Age, sex, and respondent's level of knowledge of stroke signs were among the independent predictors of intent to call 911. We found that knowledge of stroke symptoms significantly predicted intent to call 911. Thus, a cost-effective and impactful strategy to prevent stroke in similar populations could focus on addressing impediments to improving stroke literacy. For instance, stroke-related health education that is targeted toward males and the elderly (≥65 years). Since in our study, both of these factors were independently associated with a lower level of knowledge of stroke signs and symptoms or the intent to call 911 in the event of a stroke.

## Data availability statement

The original contributions presented in the study are included in the article/Supplementary material, further inquiries can be directed to the corresponding author.

## Ethics statement

The studies involving humans were approved by the Medical University of South Carolina. The studies were conducted in accordance with the local legislation and institutional requirements. The participants provided their written informed consent to participate in this study.

## Author contributions

NS: Writing—review & editing. AM: Writing—review & editing. CJ: Writing—review & editing. HH: Conceptualization, Data curation, Formal analysis, Investigation, Methodology, Resources, Supervision, Writing—original draft.
