# Supplementary material for: Predictors of stroke literacy among African Americans in the “buckle of the stroke belt”
Source: Front Stroke. 2024 Feb 27;3:1331085. doi: 10.3389/fstro.2024.1331085 (PMC12802655; doi:10.3389/fstro.2024.1331085)
Supplement: Supplementary file 1 [file Table_1.docx]

**Predictors of stroke literacy among African Americans in the ‘buckle of the stroke-belt.’**

Supplemental Table 1. Participant distribution by health behavior and health characteristics

| Variable | Weighted  Count (N) | Un-weighted count (N) | Percent (%) | 95% CI of  Percent (%) |
| --- | --- | --- | --- | --- |
| Self-described general health status  Excellent  Very Good  Good  Fair  Poor | 1642.8  2453.1  3645.2  1493.9  443.4 | 1376  2346  3800  1650  494 | 17.0  25.3  37.7  15.4  4.6 | 15.4 – 18.7  23.9 – 26.8  35.9 – 39.4  14.5 – 16.4  4.0 – 5.3 |
| Number of days of poor physical health in 30 days  None  One week or less  More than one week | 6033.4  2012.0  1608.3 | 5790  2068  1770 | 62.5  20.8  16.7 | 61.2 – 63.7  19.7 – 22.0  15.4 – 18.0 |
| Number of days of poor mental health in 30 days  None  One week or less  More than one week | 6289.0  1792.2  1592.1 | 6325  1761  1572 | 65.0  18.5  16.5 | 62.4 – 67.6  17.1 – 20.1  15.2 – 17.8 |
| Time since last routine checkup  12 months or less ago  Over 12 months ago | 7486.7  2196.9 | 7831  1844 | 77.3  22.7 | 75.6 – 78.9  21.1 – 24.4 |
| Diabetic: You check your blood sugar level regularly  Yes  No | 2359.4  701.1 | 2740  648 | 77.1  22.9 | 73.3 – 80.5  19.5 – 26.7 |
| Diabetic: You had HbA1c test in the last 12 months  Yes  No | 601.3  806.2 | 774  972 | 42.7  57.3 | 37.7 – 47.8  52.2 – 62.3 |
| Diabetic: You had feet checked in the last 12 months  Yes  No | 473.2  1198.9 | 653  1412 | 28.3  71.7 | 24.3 – 32.7  67.3 – 75.7 |
| Diabetic: You had eye exam in the last 12 months  Yes  No | 922.2  758.8 | 1075  1001 | 54.9  45.1 | 49.6 – 60.0  40.0 – 50.4 |
| Engage in Moderate activity for at least 10 minutes  Yes  No | 6947.2  2676.4 | 6891  2712 | 72.2  27.8 | 71.1 – 73.3  26.7 – 28.9 |
| Frequency of moderate intensity activities per week  3 times or less  Greater than 3 times | 3467.2  3362.4 | 3496  3278 | 50.8  49.2 | 47.8 – 53.7  46.3 – 52.2 |
| More physically active  Yes  No | 3875.9  1577.6 | 3854  1598 | 71.1  28.9 | 69.0 – 73.1  26.9 – 31.0 |
| Eating more fruits and vegetables  Yes  No | 3989.7  1463.7 | 4072  1386 | 73.2  26.8 | 71.6 – 74.7  25.3 – 28.4 |
| Job activity is mostly  Sitting or standing  Walking  Heavy labor/physically demanding | 1023.7  565.3  374.7 | 957  504  270 | 52.1  28.8  19.1 | 50.9 – 53.3  26.4 – 31.3  17.3 – 21.0 |
| Physical activity in the past month beside job  Yes  No | 2615.4  1443.0 | 2579  1475 | 64.4  35.6 | 63.5 – 65.4  34.6 – 36.5 |
| Have had cholesterol checked  Yes  No | 7467.9  2071.3 | 7949  1592 | 78.3  21.7 | 75.4 – 80.9  19.1 – 24.6 |
| Eating fewer high fat diet or high cholesterol foods  Yes  No | 3612.7  1767.3 | 3733  1642 | 67.2  32.8 | 64.7 – 69.6  30.4 – 35.3 |
| Fruits and vegetable servings consumed per day  Adequate (5+ servings)  Inadequate (<5 servings) | 2508.0  7193.9 | 2583  7122 | 25.9  74.1 | 24.0 – 27.8  72.2 – 76.0 |

Supplemental Table 2. Participant characterization by risk factor profile and their management.

| Variable | Weighted  count | Un-weighted count | Percent (%) | 95% CI of  Percent (%) |
| --- | --- | --- | --- | --- |
| Smoking risk category  Not at risk  At risk | 7640.7  1987.0 | 7887  1725 | 79.4  20.6 | 78.3 – 80.4  19.6 – 21.7 |
| Body Mass Index risk category  Not at risk  At risk | 2606.7  6787.9 | 2378  6962 | 27.7  72.3 | 26.9 – 28.6  71.4 – 73.1 |
| Been diagnosed with diabetes  Yes  No | 1731.4  7881.1 | 2136  7463 | 18.0  82.0 | 16.0 – 20.2  79.8 – 84.0 |
| Taken a course to help self-manage my diabetes  Yes  No | 510.3  271.3 | 674  363 | 65.3  34.7 | 59.2 – 70.9  29.1 – 40.8 |
| Diagnosed with high blood pressure (HBP)  Yes  No | 4289.7  5371.0 | 5020  4638 | 44.4  55.6 | 41.7 – 47.2  52.8 – 58.3 |
| Aware of anti-hypertensives and regularly taking medication  Yes  No | 3421.1  721.2 | 4191  682 | 82.6  17.4 | 80.2 – 84.7  15.3 – 19.8 |
| Changing eating habit to lower or control HBP  Yes  No | 1619.2  238.0 | 2056  267 | 87.2  12.8 | 86.1 – 88.2  11.8 – 13.9 |
| Reducing salt intake to help control or lower HBP  Yes  No  Do not use salt | 1556.6  103.9  206.8 | 1978  109  254 | 83.4  5.6  11.1 | 81.1 – 85.4  4.8 – 6.5  8.9 – 13.8 |
| Reducing alcohol to lower or control HBP  Yes  No  Do not drink alcohol | 674.8  116.6  1066.5 | 737  124  1464 | 36.3  6.3  57.4 | 33.3 – 39.5  4.4 – 8.2  52.6 – 62.1 |
| Exercising to help lower or control HPB  Yes  No | 1402.2  450.8 | 1729  585 | 75.7  24.3 | 72.6 – 78.5  21.5 – 27.4 |
| Have high blood cholesterol level  Yes  No | 2751.2  4656.8 | 3264  4615 | 37.1  62.9 | 34.4 – 40.0  60.0 – 65.6 |
| Prescribed medication to lower blood cholesterol  Yes  No | 933.1  199.3 | 1048  229 | 82.4  17.6 | 78.8 – 85.5  14.5 – 21.2 |
| Advised to reduce blood cholesterol or fat level  Yes  No | 1135.0  400.4 | 1280  424 | 73.9  26.1 | 71.1 – 76.5  23.5 – 28.9 |
| Advised to eat fewer high fat foods or lower cholesterol diet  Yes  No | 2190.3  3271.6 | 2359  3099 | 40.1  59.9 | 37.1 – 43.2  56.8 – 62.9 |
| Advised to exercise more  Yes  No | 2994.6  2497.1 | 3215  2275 | 54.5  45.5 | 50.6 – 58.4  41.6 – 49.4 |
| Advised to eat more fruits and vegetables  Yes  No | 3028.5  2455.9 | 3152  2331 | 55.2  44.8 | 53.3 – 57.1  42.9 – 46.7 |
| Have been referred to a dietician, nutritionist or nurse  Yes  No | 515.6  617.8 | 541  736 | 45.5  54.5 | 39.0 – 52.2  47.8 – 61.0 |

Supplemental Table 3. Respondents’ history of cardiovascular disease, prevention strategies, symptom awareness, intent to call 911 and prevention programs.

| Variable | Weighted  count | Un-weighted count | Percent (%) | 95% CI of  Percent (%) |
| --- | --- | --- | --- | --- |
| Doctor diagnosed heart attack or myocardial infarction  Yes  No | 337.3  9247.3 | 400  9161 | 3.5  96.5 | 3.1 – 3.9  96.1 – 96.9 |
| Doctor diagnosed angina or coronary heart disease  Yes  No | 296.1  9227.1 | 353  9131 | 3.1  96.9 | 2.7 – 3.5  96.5 – 97.3 |
| Doctor diagnosed stroke  Yes  No | 367.1  9211.4 | 413  9141 | 3.8  96.2 | 95.6 – 96.6  3.4 – 4.4 |
| Taking aspirin to reduce chance of heart attack  Yes  No | 1056.4  171.0 | 1184  200 | 86.1  13.9 | 83.0 – 88.6  11.4 – 17.0 |
| Taking aspirin to reduce chance of stroke  Yes  No | 912.6  267.7 | 1027  296 | 77.3  22.7 | 72.6 – 81.5  18.5 – 27.4 |
| Heart attack is pain or discomfort in the jaw, neck or back  Yes  No | 3772.9  5939.3 | 3856  5857 | 38.8  61.2 | 36.7 – 41.0  59.0 – 63.3 |
| Heart attack is feeling weak, light headed and faint  Yes  No | 4778.4  4935.6 | 4617  5097 | 49.2  50.8 | 47.2 – 51.2  48.8 – 52.8 |
| Heart attack is chest pain or discomfort  Yes  No | 8484.4  1229.6 | 8397  1317 | 87.3  12.7 | 86.4 – 88.2  11.8 – 13.6 |
| Heart attack is sudden trouble seeing in one or both eyes  Yes  No | 3036.6  6676.3 | 2867  6846 | 31.3  68.7 | 29.8 - 32.8  67.2 – 70.2 |
| Heart attack is pain and discomfort in the arms or shoulder  Yes  No | 7258.1  2455.1 | 7379  2334 | 74.7  25.3 | 72.7 – 76.6  27.3 – 23.4 |
| Heart attack is shortness of breath  Yes  No | 7676.0  2036.0 | 7640  2071 | 79.0  21.0 | 78.0 – 80.0  20.0 – 22.0 |
| Level of knowledge of signs and symptoms of heart attack  None to low knowledge (1 or less)  Moderate to adequate knowledge (2 or greater) | 1740.1  7969.0 | 1805  7904 | 17.9  82.1 | 16.2 – 19.8 80.2 – 83.8 |
| Stroke is sudden confusion or trouble speaking  Yes  No | 7985.9  1726.5 | 7997  1715 | 82.2  17.8 | 80.5 – 83.8  16.2 – 19.5 |
| Stroke is sudden numbness or weakness of the face, arm or leg especially on one side  Yes  No | 8711.6  1000.8 | 8671  1041 | 89.7  10.3 | 88.8 – 90.5  9.5 – 11.2 |
| Stroke is sudden trouble seeing in one or both eyes  Yes  No | 5198.5  4514.7 | 5127  4586 | 53.5  46.5 | 51.3 – 55.7  44.3 – 48.7 |
| Stroke is sudden chest pain or discomfort  Yes  No | 4130.6  5582.7 | 3927  5786 | 42.5  57.5 | 41.4 – 43.7  56.3 – 58.6 |
| Stroke is sudden trouble walking, dizziness or loss of balance  Yes  No | 7535.0  2177.7 | 7462  2250 | 77.6  22.4 | 75.7 – 79.4  20.6 – 24.3 |
| Stroke is severe headache without known cause  Yes  No | 5306.4  4406.4 | 5435  4277 | 54.6  45.4 | 52.8 – 56.4  43.6 – 47.2 |
| Level of knowledge of signs and symptoms of stroke  None to low knowledge (1 or less)  Moderate to adequate knowledge (2 or greater) | 1351.9  8359.6 | 1409  8301 | 13.9  86.1 | 12.8 – 15.1 84.9 – 87.2 |
| What would you do if you or someone were having a heart attack or stroke  Call 911 — correct response  Do anything else (call relative, taxi,  etc.) — incorrect response | 8582.8  1104 | 8637  1044 | 88.6  11.4 | 87.4 – 89.7  10.3 – 12.6 |
| Ever heard of area prevention and education program  Yes  No | 828.1  3127.8 | 901  3036 | 20.9  79.1 | 17.9 – 24.3  75.7 – 82.1 |

Supplemental Table 4. Univariate association of level of stroke knowledge, participants’ health behavior, and health characteristics.

| Variable | Odds Ratio | 95% Confidence Interval |
| --- | --- | --- |
| Self-described general health status  Excellent  Very Good  Good  Fair | 2.00  2.52  1.81  1.59 | 1.25 – 3.21  1.91 – 3.32  1.11 – 2.95  1.12 – 2.26 |
| Number of days of poor physical health in 30 days  None  One week or less | 1.23  1.50 | 1.03 – 1.45  1.21 – 1.86 |
| Number of days of poor mental health in 30 days  None  One week or less | 1.23  1.28 | 0.97 – 1.56  0.82 – 2.01 |
| Time since last routine checkup | 1.00 | 0.76 – 1.32 |
| Diabetic: You check your blood sugar level regularly | 0.80 | 0.13 – 4.91 |
| Diabetic: You had HbA1c test in the last 12 months | 0.49 | 0.37 – 0.65 |
| Diabetic: You had feet checked in the last 12 months | 1.61 | 1.07 – 2.43 |
| Diabetic: You had eye exam in the last 12 months | 1.57 | 1.13 – 2.17 |
| Engage in moderate activity for at least 10 minutes | 0.49 | 0.43 – 0.55 |
| Frequency of moderate intensity activities per week | 0.90 | 0.70 – 1.15 |
| More physically active | 0.91 | 0.65 – 1.27 |
| Eating more fruits and vegetables | 0.85 | 0.69 – 1.03 |
| Job activity is mostly  Sitting or standing  Walking | 0.93  1.23 | 0.32 – 2.70  0.27 – 5.53 |
| Physical activity in the past month beside job | 0.63 | 0.48 – 0.83 |
| Have had blood cholesterol level checked | 0.72 | 0.54 – 0.96 |
| Eating fewer high fat foods or lower cholesterol diet | 0.57 | 0.44 – 0.73 |
| Fruits and vegetable servings consumed per day | 0.91 | 0.76 – 1.09 |

Supplemental Table 5. Univariate association between stroke knowledge, participant risk factor profile and risk factor management.

| Variable | Odds Ratio | 95% Confidence Interval |
| --- | --- | --- |
| Smoking risk category | 0.81 | 0.65 – 1.01 |
| Body Mass Index risk category | 1.19 | 0.97 – 1.47 |
| Been diagnosed with diabetes | 1.28 | 1.08 – 1.51 |
| Taken a course to help self-manage my diabetes | 0.47 | 0.39 – 0.56 |
| Diagnosed with high blood pressure (HBP) | 1.13 | 1.00 – 1.28 |
| Aware of anti-hypertensives and regularly taking medication | 1.06 | 0.75 – 1.50 |
| Changing eating habit to lower or control HBP | 0.86 | 0.47 – 1.57 |
| Reducing salt intake to help control or lower HBP  Yes  No | 0.74  0.67 | 0.43 – 1.27  0.20 – 2.29 |
| Reducing alcohol to lower or control HBP  Yes  No | 1.01  0.81 | 0.79 – 1.27  0.24 – 2.76 |
| Exercising to help lower or control HPB | 1.01 | 0.87 – 1.32 |
| Have high blood cholesterol level | 1.01 | 0.90 – 1.14 |
| Prescribed medication to lower blood cholesterol | 1.01 | 0.60 – 1.68 |
| Advised to reduce blood cholesterol or fat level | 1.73 | 0.76 – 3.94 |
| Advised to eat fewer high fat foods or lower cholesterol diet | 0.63 | 0.50 – 0.79 |
| Advised to exercise more | 0.85 | 0.72 – 1.03 |
| Advised to eat more fruits and vegetables | 1.07 | 0.81 – 1.42 |
| Have been referred to a dietician, nutritionist or nurse | 0.74 | 0.41 – 1.36 |

Supplemental Table 6. Relationship between components of stroke literacy and cardiovascular literacy.

| Variable | Odds Ratio | 95% Confidence Interval |
| --- | --- | --- |
| Doctor diagnosed heart attack or myocardial infarction | 1.41 | 1.01 – 1.97 |
| Doctor diagnosed angina or coronary heart disease | 0.85 | 0.62 – 1.16 |
| Doctor diagnosed stroke | 1.32 | 0.80 – 1.61 |
| Taking aspirin to reduce chance of heart attack | 0.43 | 0.32 – 0.58 |
| Taking aspirin to reduce chance of stroke | 0.40 | 0.23 – 0.70 |
| Heart is pain or discomfort in the jaw, neck or back | 6.70 | 5.29 – 8.49 |
| Heart attack is feeling weak, lightheaded and faint | 7.70 | 5.95 – 9.97 |
| Heart attack is chest pain or discomfort | 16.81 | 13.68 -20.66 |
| Heart attack is sudden trouble seeing in one or both eyes | 7.88 | 5.35 – 11.63 |
| Heart attack is pain and discomfort in the arms or shoulder | 10.24 | 8.79 – 11.94 |
| Heart attack is shortness of breath | 11.18 | 9.35 – 13.36 |
| Level of Knowledge of signs and symptoms of heart attack | 24.16 | 19.74 – 29.56 |
| What would you do if you or someone were having a heart attack or stroke | 1.65 | 1.34 – 1.94 |
| Ever heard of area prevention and education program | 0.76 | 0.54 – 1.09 |
